# First evidence of a large *CHEK2* duplication involved in cancer predisposition in an Italian family with hereditary breast cancer

**DOI:** 10.1186/1471-2407-14-478

**Published:** 2014-07-01

**Authors:** Gianluca Tedaldi, Rita Danesi, Valentina Zampiga, Michela Tebaldi, Lucia Bedei, Wainer Zoli, Dino Amadori, Fabio Falcini, Daniele Calistri

**Affiliations:** 1 Istituto Scientifico Romagnolo per lo Studio e la Cura dei Tumori (IRST) IRCCS, Meldola, Italy; 2 Cancer Prevention Unit, Morgagni-Pierantoni Hospital, Forlì, Italy

**Keywords:** *CHEK2*, Duplication, Breast cancer, Hereditary cancer, MLPA, Next-generation sequencing

## Abstract

**Background:**

*CHEK2* is a multi-cancer susceptibility gene whose common germline mutations are known to contribute to the risk of developing breast and prostate cancer.

**Case presentation:**

Here, we describe an Italian family with a high number of cases of breast cancer and other types of tumour subjected to the MLPA test to verify the presence of *BRCA1*, *BRCA2* and *CHEK2* deletions and duplications. We identified a new 23-kb duplication in the *CHEK2* gene extending from intron 5 to 13 that was associated with breast cancer in the family. The presence and localisation of the alteration was confirmed by a second analysis by Next-Generation Sequencing.

**Conclusions:**

This finding suggests that *CHEK2* mutations are heterogeneous and that techniques other than sequencing, such as MLPA, are needed to identify *CHEK2* mutations. It also indicates that *CHEK2* rare variants, such as duplications, can confer a high susceptibility to cancer development and should thus be studied in depth as most of our knowledge of *CHEK2* concerns common mutations.

## Background

Breast cancer is the most frequent invasive cancer in women and its incidence is increasing in Western countries [1]. Hereditary breast cancer (HBC) represents only 5-10% of all breast cancer cases. The principal genes involved in the risk of breast cancer are *BRCA1* and *BRCA2*, which account for about 20-50% of all HBC cases. To date, other low-penetrance genes, correlated with the risk of breast cancer, have been identified, such as *ATM*, *BRIP1*, *CDH1*, *PALB2* and *CHEK2*[2].

The checkpoint kinase 2 gene (*CHEK2*, MIM# 604373) is located in 22q12.1 and is the human homolog of RAD53 (*Saccharomyces cerevisiae*) and CDS1 (*Schizosaccharomyces pombe*).

CHEK2 protein is located in the nucleus and consists of 543 amino acids with three main domains: a SQ/TQ cluster domain (aa 20–75), a forkhead-associated domain (aa 115–165) and a Ser/Thr kinase domain (aa 225–490) [3].

In response to DNA double-strand breaks, CHEK2 protein is phosphorylated by ATM and catalyses the phosphorylation of CDC25C, down-regulating it and preventing entry into mitosis [4].

Furthermore, after DNA damage, CHEK2 phosphorylates the p53 tumour suppressor protein and prevents its degradation, leading to cell cycle arrest in G1 [5].

Under gamma irradiation, CHEK2 also phosphorylates BRCA1 on Ser-988, activating the DNA repair process [6]. Finally, CHEK2 has been shown to induce apoptosis independently of p53, via phosphorylation of the PML tumour suppressor protein [7].

The first germline mutations identified in the *CHEK2* gene were associated with Li-Fraumeni syndrome (MIM# 609265), a disease characterised by high occurrences of sarcomas, breast cancer, brain tumours, acute leukaemia and adrenocortical tumours [8].

Bell identified *CHEK2* mutations in three families, including the mutation 1100delC in the kinase domain, resulting in premature termination [9].

This cytosine deletion at nucleotide 1100 of *CHEK2* sequence was also identified in two Finnish families with atypical Li-Fraumeni syndrome due to the lack of sarcomas or childhood cancers [10].

Subsequently, the same mutation was identified in *BRCA1*/*2*-negative families with hereditary breast cancer and it has been estimated that it results in an approximately two-fold increase of the risk of breast cancer in women and a ten-fold increase of risk in men [11]. The 1100delC mutation shows a high prevalence in northern Europe and its homozygosity confers a fourfold increased risk of breast cancer in women [12].

Dong found 28 *CHEK2* germline mutations among 578 men with prostate cancer, suggesting that *CHEK2* mutations play a role in prostate cancer development [13], and Cybulski found common mutations, such as 1100delC, IVS2 + 1G > A and I157T, in families with hereditary prostate cancer [14].

More recently, common mutations in *CHEK2*, such as 1100delC, I157T and IVS2 + 1G > A, have been associated with different types of cancer [15-17], but the association of *CHEK2* mutations with Li-Fraumeni syndrome has been questioned because of different phenotypes in Li-Fraumeni patients and *CHEK2* mutation carriers [18].

In 2006, a novel 5.6 kb deletion of exons 9 and 10 was discovered in two families of Czechoslovakian ancestry with hereditary breast and ovarian cancer and the alteration has also been detected in 8 out of 631 patients with breast cancer and in none of the 367 controls from the Czech and Slovak Republics [19]. Cybulski and co-workers also detected this deletion in 15 out of 1864 males with unselected prostate cancer and in 4 out of 249 males with familial prostate cancer and concluded that this alteration doubles the risk of prostate cancer in men and quadruples the risk of familial cases [20]. However, this large deletion seems to be a founder mutation present only in Slavic populations.

Recently, in France, 16 *CHEK2* mutations were found in 507 cases of *BRCA1*/*2*-negative hereditary breast cancer. Nine of these variants were novel and no mutation hotspots were identified, suggesting that *CHEK2* mutations are spread throughout the gene and that *CHEK2* mutation screening in populations where the common founder mutations are rare must consider the entire coding region [21]. To date, less than 100 mutations in the *CHEK2* gene have been identified: the Human Gene Mutation Database *v. Professional 2013.4* contains 55 missense/nonsense mutations, 2 gross deletions, 11 small deletions, 1 small insertion, 3 splice-site mutations and 2 mutations in regulatory regions.

## Case presentation

**Figure 1:**
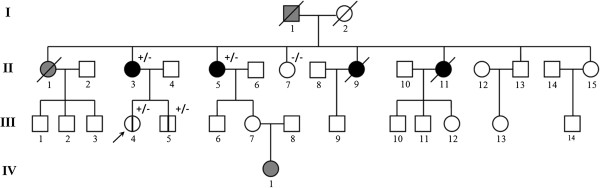
**Pedigree of the family with duplication in *CHEK2.*** Black symbols indicate breast cancer and grey symbols indicate other type of tumours (see text); the proband is indicated by an arrow; members of the family submitted to the genetic test are indicated by +/− for mutation carriers or −/− for non-mutation carriers; the vertical bar indicates unaffected mutation carriers.

We report the identification of the first large duplication in the *CHEK2* gene in a family with a history of breast cancer, detected during a study on hereditary breast cancer. The study was performed in accordance with the principles of Good Clinical Practice and the ethical standards laid down in the Declaration of Helsinki and approved by the IRST Ethical Committee (CE IRST IRCCS-AVR, protocol 2207/2012).The first family member who came to our attention through our regional screening protocol for breast cancer was a woman aged 36 (III-4) who did not have cancer but had a high family incidence of breast cancer (Figure 1). Her mother (II-3) had bilateral breast cancer at 46 years old and 56 years old; three aunts had breast cancer at 70 years old (II-5), 50 years old (II-9) and 40 years old (II-11); and another aunt died of ovarian cancer at 40 years old (II-1). The grandfather of the proband (I-1) died of prostate cancer at 80 years old. Furthermore, the brother of the proband (III-5) has a clinical suspicion of neurofibromatosis for which he is under investigation and a cousin’s daughter (IV-1) developed leukaemia at 3 years old.

The proband III-4 was subjected to mutation analysis of *BRCA1*/*2*: after her informed consent was obtained, we collected peripheral blood of III-4 and we extracted the DNA from leukocytes using the QIAamp DNA mini kit (Qiagen, Hilden, Germany). All the coding exons and flanking introns of *BRCA1* and *BRCA2* were amplified by PCR with Ex Taq DNA polymerase (Takara, Otsu, Japan) and subjected to Sanger sequencing using the BigDye® Terminator v3.1 (Life Technologies, Carlsbad, CA). The sequences were analysed by capillary electrophoresis on the 3130 Genetic Analyzer (Life Technologies).

The *BRCA1*/*2* mutation test on III-4 detected a number of variants in *BRCA1* that were classified as polymorphisms on the basis of the Universal Mutation Database (http://umd.be/BRCA1/): c.2082C > T (rs1799949), c.2311 T > C (rs16940), c.2612C > T (rs799917), c.3113A > G (rs16941) in exon 11, c.4308 T > C (rs1060915) in exon 13 and c.4837A > G (rs1799966) in exon 16.

The same polymorphisms had also been found in her mother (index case), who had undergone the *BRCA1*/*2* mutation test at another laboratory.

These variants could not explain the high familiarity of breast cancer in this family and we, therefore, conducted on III-4 the Multiplex Ligation-dependent Probe Amplification analysis (MLPA) for deletions/duplications of all *BRCA1*/*2* exons with BRCA1-P002-C2 and BRCA2-P045-B3 (MRC Holland, Amsterdam, the Netherlands).

In particular, the BRCA2-P045 kit contains probes for all the exons of *BRCA2* and three additional probes for the *CHEK2* gene: a probe for the promoter region, a probe specific to the 1100delC mutation [9] and a probe for exon 9, whose deletion has been detected in families with hereditary prostate and breast cancer [19,20].

The samples were loaded onto the 3130 Genetic Analyzer (Life Technologies) and the results were analysed using Coffalyser.net software (MRC-Holland). The software divides the normalised peak areas of the patient by the average peak areas of reference samples (four reference samples from healthy individuals were introduced in each run). The software gives a value for each exon that is normal between 0.7 and 1.3 and indicates a deletion under 0.7 or a duplication over 1.3. The analysis of the results with Coffalyser.net showed a duplication (value of 1.68) for the exon 9 of *CHEK2* in patient III-4 (data not shown).

**Figure 2:**
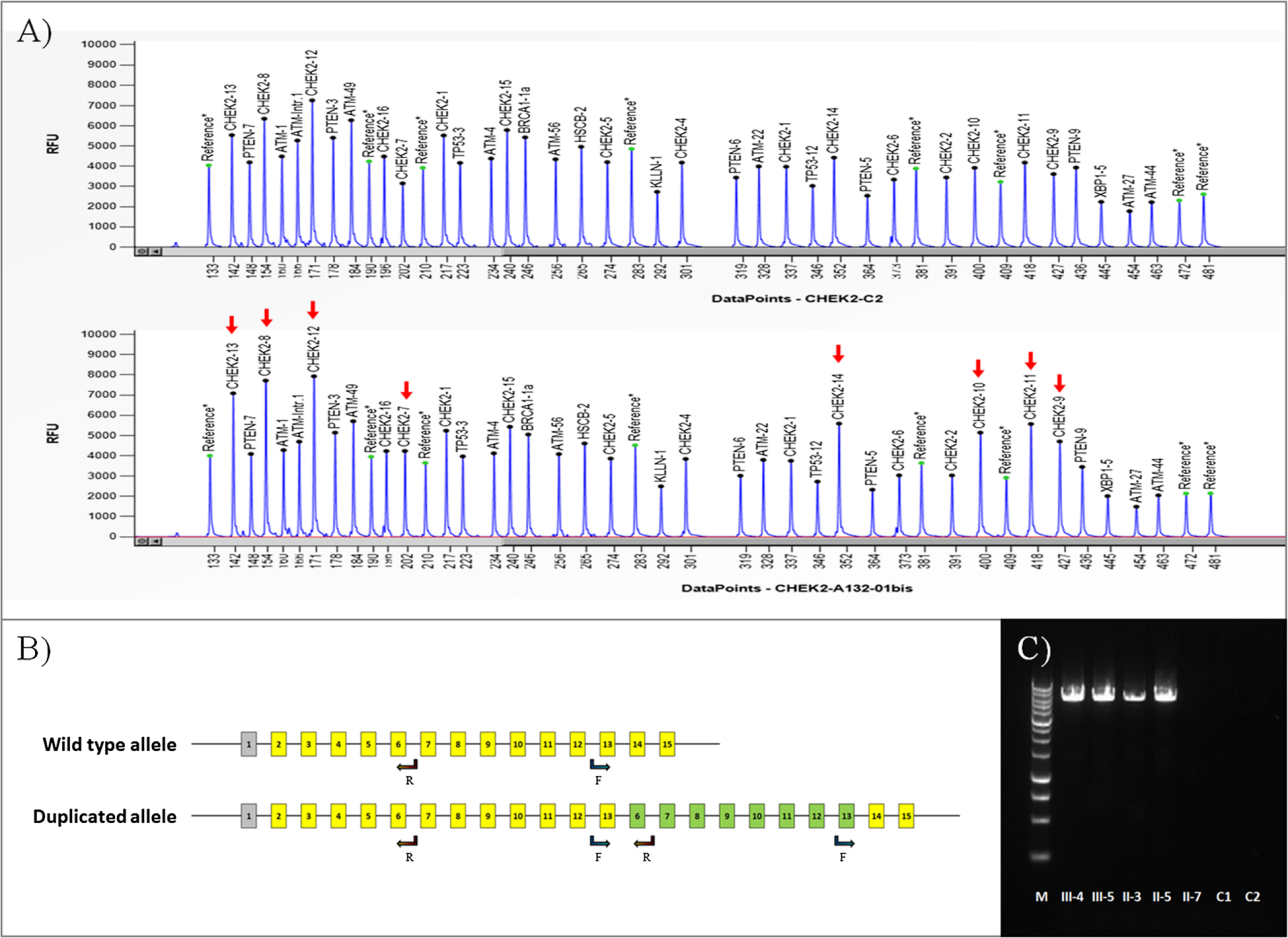
**Results of the molecular analysis on the *CHEK2 *duplication. (A)** Electropherograms showing the MLPA probes for *CHEK2* exons in a reference sample (upper panel) and in proband III-4 (lower panel): duplicated exons are indicated by red arrows (Coffalyser software uses the NM_001005735.1 transcript of *CHEK2*). **(B)** Schematic representation of *CHEK2* gene showing the wild type allele and the duplicated allele (transcript NM_007194.3 with non-coding exon 1 in grey): arrows indicate primers 13-forward and 6-reverse used for PCR. **(C)** Agarose gel showing the breakpoint of the duplication amplified by PCR: the reaction generated a 6-kb product in patients with the duplication (II-3, II-5, III-4 and III-5) but not in the wild type individual (II-7) or negative controls (C1 and C2). M, molecular weight size marker 1 kb (Promega).

In order to confirm the data and to verify the extension of the duplication, we performed the analysis of the sample with the CHEK2-P190-B1 kit (MRC Holland), which contains probes for each *CHEK2* exon. The new analysis with Coffalyser.net showed that the duplication extends from exon 6 to exon 13 (NM_007194.3) with ratios of 1.29-1.51 (Figure 2A).

The MLPA analysis of *CHEK2* was extended to four other members of the family who, after providing their informed consent, agreed to participate in the test (II-3, II-5, II-7 and III-5 in Figure 1). The mother of the proband (II-3) and her aunt (II-5), both of whom had had breast cancer, had the same duplication in *CHEK2*. The brother of the proband (III-5), who had multiple subcutaneous cysts similar to neurofibromas, had the same duplication as the sister. The aunt II-7, who had never had cancer, did not have the duplication.In order to confirm the duplication and its exact size (the MLPA kits used in our experiments do not provide information on introns) with another method, the region containing the breakpoint was amplified. For this purpose, we designed a primer forward that anneals before exon 13 (5’-GTCTGCTGACTCCGTGATGA) and a primer reverse that anneals after exon 6 (5’-TGGGGTTACAGTGGGGATTA). Due to their specific design, these two primers only generate a PCR product in patients who have the duplication and do not generate any product in the absence of duplication (Figure 2B).The PCR products were analysed by agarose electrophoresis and the ethidium bromide staining showed a fragment of about 6 kb in size, present only in DNA from patients with the duplication and not in wild type individuals and in normal human DNA, used as a control (Figure 2C).

In order to verify the exact position of the breakpoint, we sequenced the amplicon by loading it onto the Next-Generation Sequencing platform Miseq (Illumina, San Diego, CA) using the Nextera XT kit (Illumina). The analysis of the results with Miseq Reporter 2.2.29 software (Illumina) showed that the region duplicated was almost 23 kb and that it extended from intron 5 at position 29111154 to intron 13 at position 29088207 (NCBI RefSeq: NC_000022.10).

## Conclusions

In this study, we report the identification of the first large germline duplication in *CHEK2* gene. This finding suggests that *CHEK2* alterations could be more heterogeneous than expected and that it is important also to perform the research for duplications and deletions of *CHEK2* gene by techniques such as MLPA, as these alterations cannot be detected by direct sequencing.

The presence and localisation of the duplication was confirmed by a second method, reinforcing the fact that this duplication was not an artefact caused by the presence of *CHEK2* pseudogenes [22]. We evaluated the effect of the duplication and found that it created a frameshift at codon 488 in the kinase domain involved in the interaction of the protein with its targets and in the activation of the repair process after DNA damage, resulting in a premature termination at codon 503. The alteration reported here has not been detected in any of the other 244 *BRCA1*/*2*-negative patients with a family history of breast and ovarian cancer analysed with MLPA in our laboratory, suggesting that this duplication is a rare variant and it is conceivable that it could contribute in an appreciable manner to cancer development.

Although we identified the *CHEK2* duplication in a family with a prevalence of breast cancer, two of the carriers of the duplication were unaffected individuals (III-4 and III-5 in Figure 1). All of the patients with the *CHEK2* duplication have been placed on a surveillance protocol that differs according to age and sex: the young unaffected woman (III-4 in Figure 1) is monitored by breast ultrasound and clinical examination every 6 months and by mammography every two years, while her mother and aunt (II-3 and II-5 in Figure 1) have been placed on a different surveillance protocol that comprises an annual breast ultrasound and mammography. Her unaffected brother (III-5 in Figure 1) is being monitored for prostate and breast cancer and undergoes annual PSA testing and breast ultrasound.

Some family members had different tumours, such as prostate cancer, ovarian cancer and leukaemia. Unfortunately, it was not possible to conduct the test on these patients to confirm the association of the duplication with these tumours because they had already died or had not reached the legal age for genetic testing.

*CHEK2* has been described as a low-penetrance gene because its inherited mutations appear less severe, leading to an increase in the risk of breast cancer in women [11,12] and prostate cancer in men [13,14,20]. These observations refer to commonly identified mutations but this case report appears to suggest that rare *CHEK2* mutations, such as large duplications, can also be detected and could determine a higher than expected increase in the risk of cancer. For this reason, further studies are required in order to investigate the mechanisms by which rare mutations in *CHEK2* participate in tumour development.

### Consent

Written informed consent was obtained from the patients for publication of this Case report and any accompanying images. A copy of the written consent is available for review by the Editor of this journal.

## Abbreviations

HBC: Hereditary breast cancer; Aa: Amino acid; SQ/TQ: Serine-glutamine/treonine-glutamine; MLPA: Multiplex ligation-dependent probe amplification.

## Competing interests

The authors declare that they have no competing interests.

## Authors’ contributions

GT carried out the molecular genetic studies, was involved in all steps of data analysis and manuscript writing; RD was involved in the patient recruitment and in the clinical follow-up and helped to draft the manuscript; VZ was involved in the data analysis and helped to draft the manuscript; MT provided laboratory support in molecular genetic studies; LB was involved in patient recruitment and provided clinical data; WZ was involved in the conception and design of the study and critically reviewed the manuscript; DA and FF were involved in the conception and design of the study; DC was directly involved in the conception and design of the study, supervised all the steps of the analysis and coordinated manuscript writing. All authors read and approved the final manuscript.

## Pre-publication history

The pre-publication history for this paper can be accessed here:

http://www.biomedcentral.com/1471-2407/14/478/prepub
